# Socioeconomic and environmental predictors of estuarine shoreline hard armoring

**DOI:** 10.1038/s41598-019-52504-y

**Published:** 2019-11-08

**Authors:** Nicole E. Peterson, Craig E. Landry, Clark R. Alexander, Kevin Samples, Brian P. Bledsoe

**Affiliations:** 1 Graduate Research Assistant, School of Environmental, Civil, Agricultural, and Mechanical Engineering, Institute for Resilient Infrastructure Systems (IRIS), University of Georgia, Athens, GA 30602 USA; 2 EA Engineering, Science, and Technology, Inc., PBC, 320 Gold Avenue Southwest, Suite 1300, Albuquerque, NM 87102 USA; 3 Professor, Department of Agricultural and Applied Economics, IRIS, University of Georgia, 0301 Conner Hall, 147 Cedar Street, Athens, GA 30602 USA; 4 Director and Professor, Skidaway Institute of Oceanography, Department of Marine Science, IRIS, University of Georgia, 10 Ocean Science Circle, Savannah, GA 31411 USA; 5 GIS Analyst, IRIS, School of Environmental, Civil, Agricultural, and Mechanical Engineering, University of Georgia, 0712L Boyd Graduate Research Building, 200 D.W. Brooks Drive, Athens, 30602 USA; 6 Director and Professor, IRIS, School of Environmental, Civil, Agricultural, and Mechanical Engineering, University of Georgia, 0712M Boyd Graduate Research Building, 200 D.W. Brooks Drive, Athens, GA 30602 USA

**Keywords:** Environmental impact, Environmental social sciences

## Abstract

Rising sea levels and growing coastal populations are intensifying interactions at the land-sea interface. To stabilize upland and protect human developments from coastal hazards, landowners commonly emplace hard armoring structures, such as bulkheads and revetments, along estuarine shorelines. The ecological and economic consequences of shoreline armoring have garnered significant attention; however, few studies have examined the extent of hard armoring or identified drivers of hard armoring patterns at the individual landowner level across large geographical areas. This study addresses this knowledge gap by using a fine-scale census of hard armoring along the entire Georgia U.S. estuarine coastline. We develop a parsimonious statistical model that accurately predicts the probability of armoring emplacement at the parcel level based on a set of environmental and socioeconomic variables. Several interacting influences contribute to patterns of shoreline armoring; in particular, shoreline slope and the presence of armoring on a neighboring parcel are strong predictors of armoring. The model also suggests that continued sea level rise and coastal population growth could trigger future increases in armoring, emphasizing the importance of considering dynamic patterns of armoring when evaluating the potential effects of sea level rise. For example, evolving distributions of armoring should be considered in predictions of future salt marsh migration. The modeling approach developed in this study is adaptable to assessing patterns of hard armoring in other regions. With improved understanding of hard armoring distributions, sea level rise response plans can be fully informed to design more efficient scenarios for both urban development and coastal ecosystems.

## Introduction

The co-occurrence of sea level rise (SLR) and expanding coastal populations creates shifting, often overlapping, boundaries between intertidal ecosystems and the built environment, increasing the potential for conflict and competition for the same space on the coastline^1^. Global average sea level has been rising at an accelerated rate during the last several decades, primarily due to melting continental ice sheets and glaciers, in addition to ocean thermal expansion and changes in terrestrial water storage^2–4^. Projections for the year 2100 bound global mean SLR between 0.3 and 2.5 m above the 1992 mean sea level^5^, indicating an inevitable increase in the intrusion of ocean waters into developed coastal areas. Despite impinging water levels, coastal populations are growing substantially^6^, even in areas where significant land loss to water has already occurred^7^.

As sea level and developed lands converge, coastal communities and residents have three general response options: protect, accommodate, or retreat^8–10^. The ‘protect’ option has been historically dominant and typically refers to the use of hard armoring structures such as bulkheads and revetments to fortify the existing upland-estuarine boundary and preserve dry land^11^. While ‘soft’ and ‘hybrid’ protection designs that focus on the incorporation of nature-based features have recently garnered attention for simultaneously providing coastal resiliency and a range of ecological functions^12,13^, hard armoring continues to increase along populated coastal shorelines^14–16^.

Improved understanding of the extent, drivers, and consequences of hard armoring implementation is needed to inform societal response to SLR. While hard armoring may provide immediate protection benefits to coastal landowners, these structures can negatively affect coastal ecosystems by contributing to the disturbance and loss of habitat^17–20^. A particularly important effect of hard armoring structures has been termed “coastal squeeze,” i.e., the bounding of coastal marshes between rising sea levels and hard armoring that prevents salt marsh migration to higher elevations^21,22^, ultimately leading to loss of habitat and associated ecosystem services^23,24^. In addition to providing unique and critical natural habitat, coastal salt marshes provide valuable services to humans, including provisioning of food, maintenance of fisheries, water purification^25^, carbon sequestration^26^ and coastal erosion and flood protection^27–29^. Hard armoring abundance and configuration may be the dominant factor influencing the future extent of coastal salt marshes in some locations^30–34^.

Continued implementation of hard armoring will induce significant financial impacts. For example, the costs of adapting to the joint effects of SLR and storm surge in the United States are estimated to be an undiscounted $990 billion through 2100 under a mid-range climate-sensitivity scenario; the largest share of this projected cost is associated with implementation of hard armoring^35^. Due to the imminent threats to coastal communities posed by SLR (and in some places, land subsidence), developing SLR response strategies that consider environmental and socioeconomic repercussions is an urgent need. Knowledge regarding the current state of SLR response tactics is likely to be foundational in the development of future response plans and policies.

This study addresses a knowledge gap in the documentation and understanding of hard armoring patterns and their drivers at the scale of individual landowners. A fully informed SLR response strategy, including evaluation of socioeconomic and environmental impacts, requires a comprehensive inventory of existing shoreline armoring structures and a fundamental understanding of the factors that influence developmental patterns of shoreline armoring at the local scale, as effective SLR response strategies will necessarily be tailored to local contexts^36^. Previous studies have investigated the decision making processes of coastal resident response to SLR by identifying potential drivers of hard armoring at the individual homeowner^37–39^ and county^40^ scales. These studies collectively suggest that contextual and scale-dependent factors are associated with patterns of hard armoring. The individual-level studies, however, relied on the use of geographically focused surveys^37–39^, thus suffering from limited participation and response bias. The county-level study, alternatively, presents a coarse-grained analyses using aggregated socioeconomic and environmental attributes^40^, thus omitting site specific features and neglecting differences between a county’s inland and shoreline characteristics.

Here we take several steps to assess and understand patterns of hard armoring at the parcel-level scale along the entire Georgia estuarine coastline using a new methodology. First, we develop a list of socioeconomic and environmental attributes that we hypothesize to be associated with the presence or absence of hard armoring. We then assign quantitative representations of these attributes to individual parcels and use a novel, fine-scale census of hard armoring along the Georgia coast to identify individual armored and unarmored parcels; given the abundance of salt and brackish marsh habitats that may be affected by current and future hard armoring emplacement, we solely assess parcels abutting estuarine shoreline. Finally, we perform logistic regression analysis to test our hypothesized relationships and examine the power of the socioeconomic and environmental attributes in predicting emplacement of estuarine shoreline hard armoring.

## Methods

### Defining the study area

**Figure 1:**
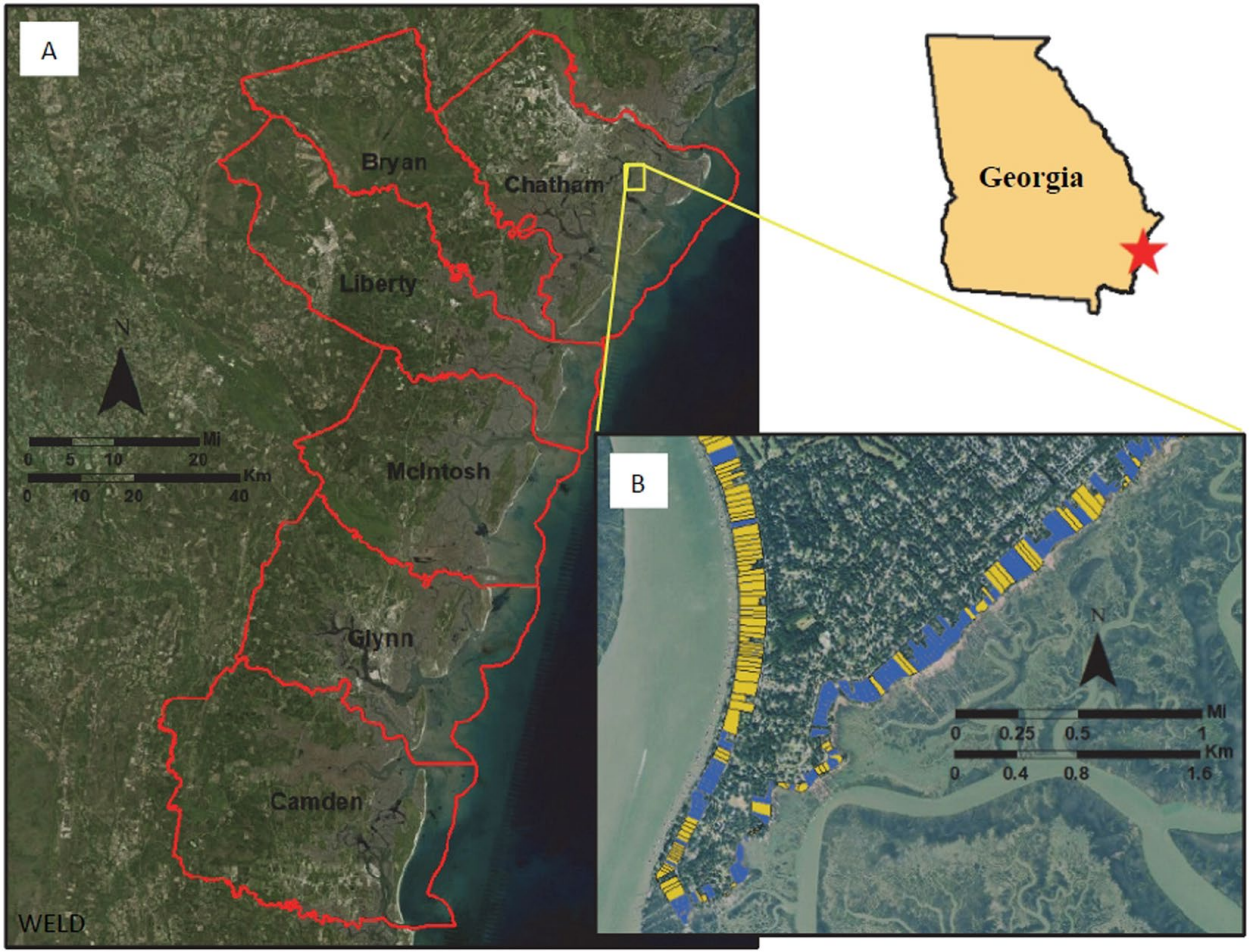
The study area is defined by estuarine shoreline parcels within the six Georgia coastal counties: (**A**) delineations of each of the six Georgia coastal counties and (**B**) delineations of individual estuarine shoreline parcels where blue and yellow indicate armoring absence and presence, respectively. Images were generated in ArcGIS Desktop 10.5 using NASA’s Web-Enabled Landsat Data (WELD) (10.5067/MEaSUREs/WELD/WELDUSYR.001) for (**A**) and USDA 2017 NAIP Digital Ortho Photo Imagery (10.5066/F7QN651G) for (**B**).

The geographical domain of this study is defined within the six counties that comprise the Georgia coastline: Camden, Glynn, McIntosh, Liberty, Bryan, and Chatham (Fig. 1). The Georgia coastline is approximately 100 miles long and includes thirteen barrier islands and nine major estuaries. A defining characteristic of the Georgia coastline is its vast expanse of salt marshes situated in estuarine environments. In comparison to other U.S. coastlines, the Georgia coastline is largely undeveloped. Accordingly, it is home to nearly one-third of all salt marshes along the U.S. Atlantic coastline^41^. Previous research has established that approximately 92% of Georgia’s estuarine shoreline is solely or dominantly fronted by salt marsh and approximately 5% of the shoreline is armored^42^.

This study was performed at the scale of individual land parcels. Parcel boundaries and parcel-level tax assessor information were provided by the Coastal Regional Commission (CRC) of Georgia and referenced 2016 computer assisted mass appraisal (CAMA) data. Using ArcMap 10.3.1, we specifically identified shoreline parcels for inclusion in our analysis, as these landowners are directly facing the decision of whether to armor their shoreline. We defined shoreline parcels to be those with dry land (upland) abutting wetland or water habitat. To identify shoreline parcels, we first used the National Wetlands Inventory (NWI)—Estuarine and Marine Deepwater and the NWI—Estuarine and Marine Wetland Habitats^43^ to create a polyline delineating the coastal shoreline; these NWI habitats approximately encompass areas of salt marsh, brackish marsh, estuarine open water, and open ocean. This polyline was converted to a 1-m grid and then to points. To select shoreline parcels and discard non-shoreline parcels, we overlaid the delineation of the CAMA parcel boundaries with the shoreline points and used the ‘Near’ function to identify the nearest parcel to each point; these parcels were denoted as shoreline parcels. The inland extent of our analysis is bounded by the westernmost extent of either I-95 or U.S. Highway 17, as this is the inland extent of the Georgia Coast Armored Shoreline dataset coverage^14^.

Based on visual quality assurance checks on the identification of shoreline parcels, we implemented two refinements to our methodology that improved shoreline parcel identification for our purposes. The first addressed our finding that the CAMA data included some multi-part parcels consisting of fully inland, disconnected polygons in addition to polygons associated with shoreline property. To remove the inland parcel areas from our analysis, we used ArcMap to separate all multi-part parcels into multiple single parcels before identifying parcels with shoreline property. The second refinement addressed our finding that some parcel boundaries extended seaward of the shoreline polyline, into a wetland or water area. Because we were interested in determining the dry land area of each parcel for future use in the analysis, we used ArcMap to clip parcel boundaries to the shoreline polyline. Upon creation of these new parcel boundaries, we identified numerous parcels with little of their original area remaining. Upon visual inspection, a majority of these parcels were found to be irregularly shaped delineations of predominantly water and wetland influenced areas. These areas acted to prevent the selection of the desired shoreline parcels; thus, we removed parcels with <10% of their original area from the entirety of the analysis. Shoreline parcels were reassessed after the removal of these parcel fragments.

We subsequently classified the shoreline type for each parcel to be either ‘estuarine’ or ‘marine’ based on whether the centroid of the parcel was nearer the NWI Estuarine and Marine Deepwater sub-classification of E1UBL (Estuarine, Subtidal, Unconsolidated Bottom, Subtidal) or M1UBL (Marine, Subtidal, Unconsolidated Bottom, Subtidal)^43^, respectively. Due to the potential for hard armoring to influence salt marsh habitat and migration, we solely included parcels with estuarine shoreline in our analysis. Parcels selected based on the above specified criteria represent a census of estuarine shoreline parcels along the Georgia coastline.

### Identifying armored parcels

We used a novel, high-resolution dataset^14,﻿15﻿^ to define the distribution of hard armored shorelines throughout the estuarine area of Georgia at the parcel level. Using aerial imagery from 2006 and 2013, combined with extensive field inspection efforts, this dataset identifies the type (bulkhead, revetment, bulkhead and revetment, road causeway, other, unknown) and location of hard armoring structures, using coordinates to define these features as polylines in a Geographic Information System. The armoring structures here have largely been implemented for erosion control purposes, with bulkheads and revetments constituting a majority of the armoring structures (>85%)^14,15^. We did not include road causeways in our analysis because we sought to understand drivers of shoreline armoring emplaced by landowners at the parcel scale. ‘Soft’ armoring approaches such as living shorelines were not considered in this study, as these techniques were outside the scope of the research investigation and their current use is rare along the Georgia coastline.

Methods for identifying parcels with armored shorelines paralleled the approach used to identify shoreline parcels. Armoring polylines were converted into a 1-m grid and then converted into points in ArcMap. CAMA parcel boundaries were then overlaid with these points and the ‘Near’ function was used to identify the nearest parcel to each point; these parcels were coded as being armored. Visual quality assurance checks on associations between individual parcels and armoring structures led us to redefine armored parcels as parcels with an armoring length >25% of their shoreline length. Thus, we estimated the armoring and shoreline lengths for each parcel based on the number of armoring and shoreline points associated with the parcel. We recoded all parcels with an armoring length <25% of their shoreline length as unarmored.

### Attribute selection and corresponding data collection

Through literature review^37,39,40^, application of microeconomic behavioral theory^9,10,44^, consideration of the three components of vulnerability (exposure, adaptive capacity, and sensitivity^45,46^), and application of local knowledge based on field reconnaissance and informal elicitation of landowner perspectives, we developed a refined list of socio-economic and environmental attributes that we hypothesized to be associated with the presence or absence of estuarine shoreline hard armoring at the parcel level scale along the Georgia coastline (Table 1). For each attribute that we identified, it was necessary for us to obtain parcel-level information representing the attribute of interest either directly or indirectly. We pursued datasets that provided information for the largest number of parcels along the Georgia coastline, and at a scale appropriate for parcel level analysis. We then used descriptor variables to numerically or categorically describe each attribute; in some instances, we identified several descriptor variables to serve as proxies for complex characteristics. Some descriptor variables were directly provided in a dataset at the parcel scale while others required calculation and/or manipulation of a dataset in ArcMap.

**Table 1:** Attributes and corresponding descriptor variables hypothesized to be associated with the presence (+) or absence (−) of hard armoring.

Attribute and Source	Descriptor Variable	Relationship Hypothesis	Description (methodology used for evaluation)
Distance to Shoreline^43^	Distance to Shoreline (m)	−	Shortest distance from the centroid of the parcel area to the shoreline polyline
Elevation^56,57^	Elevation (m)	−	Mean elevation of parcel area relative to the North American Vertical Datum of 1988 (NAVD88)
Slope	Elevation/Distance To Shoreline	+	Ratio of elevation to distance (each defined above)
Parcel Area (CRC of Georgia)	Parcel Area (km^2^)	+	Upland area of the parcel
Shoreline Energy Class^43^	Indicators for Low, Medium, and High Energy	+	Classification of shoreline energy based on shoreline type from the NWI classification
Shoreline Change^58^^*^	Minimum Historical Shoreline Change Rate (m/year)	−	Minimum value of the shoreline change rate transects that overlap with the original CAMA parcel boundary
	Average Historical Shoreline Change Rate (m/year)	−	Average value of the shoreline change rate transects that overlap with the original CAMA parcel boundary
	Maximum Historical Shoreline Change Rate (m/year)	−	Maximum value of the shoreline change rate transects that overlap with the original CAMA parcel boundary
	Erosion Rate (m/year)	+	Absolute value of the average historical shoreline change rate descriptor variable for values <0
	Accretion Rate (m/year)	−	Value of the average historical shoreline change rate descriptor variable for values >0
Influence of Neighboring Armor^14,15^	Neighbor Armoring (binary)	+	Denotes if a parcel adjoins another parcel that has hard armoring (1) or not (0)
	Distance to Closest Armored Neighbor (m)	−	Distance from the centroid of a parcel area to the centroid of the closest armored parcel area, other than the parcel itself
Parcel Value (CRC of Georgia)	Replacement Cost ($)	+	Replacement cost for buildings on parcel
	Construction Cost ($)	+	Construction cost for buildings on parcel
	Building Area (m^2^)	+	Area of buildings on parcel
	Total Value ($)	+	Total value of parcel
Urban Classification^59^	Housing Unit Density at the Block Scale (hu/km^2^)	+	Housing unit count for the block in which a parcel is located, divided by the area (m^2^) of that block as given in the census data
	Population Density at the Block Scale (ppl/km^2^)	+	Population count for the block in which a parcel is located, divided by the area (m^2^) of that block as given in the census data
	Housing Unit Density at the Block Group Scale (hu/km^2^)	+	Housing unit count for the block group in which a parcel is located, divided by the area (m^2^) of that block group as given in the census data
	Population Density at the Block Group Scale (ppl/km^2^)	+	Population count for the block group in which a parcel is located, divided by the area (m^2^) of that block group as given in the census data

*The data source for the shoreline change attribute (58) applies negative numbers to rates of erosion and positive numbers to rates of accretion.

All estuarine shoreline parcels were assigned a value for each descriptor variable. In some instances, this required the value of zero to be assigned to parcels with null or missing values. Specifically, the descriptor variables of replacement cost, construction cost, and building area were assigned a value of zero if specified as null in the CAMA data provided by the CRC of Georgia. We determined this methodology to be appropriate for our analysis because a null value indicates an absence of buildings on a parcel, and we are interested in capturing the value of buildings on the parcel through these descriptor variables. The descriptor variables for the shoreline change attribute were also assigned values of zero when historical shoreline change transects did not overlap the original CAMA parcel boundary. The historical shoreline change rate dataset documents the historical rate of shoreline change between ca. 1930–2010 at 50 m intervals along the shore, and in some instances, locations where historical shoreline change rates were assessed occurred outside of the original CAMA parcel boundaries. Historical shoreline change rates were evaluated along prominent waterways, leaving parcels abutting smaller tidal creeks and marshes at the inland extent of the study area without a measure of historical shoreline change. Only limited data were available for smaller creek systems and the naturally meandering nature of these creeks (e.g., erosion on one bank balanced by accretion on the other) makes it impossible to appropriately generalize rates for these systems^47^. We determined that it was most appropriate to estimate the shoreline change values to be zero for parcels that were not associated with a historical shoreline change rate after we applied the methods specified above (Table 1), as these areas are situated in low energy environments where rates of change are low in comparison to ocean front and open fetch settings. We performed extensive spot-checking of all descriptor variables, leading us through several iterations of refining ArcMap commands and calculations.

### Logistic regression analysis

We performed logistic regression analysis using Stata 15^48^ to probabilistically assess the spatial distribution of hard armoring as a function of select descriptor variables (Table 1); the logistic regression model estimates can be used to provide a probability of hard armoring (0 < *p* < 1) on a specified parcel. Model development was directed towards capturing the influential factors in the individual decision to invest in hard armoring (or purchase properties that already had armoring installed). The general form of our model was based on conceptual choice theory, as we viewed the probability of installing hard armoring as a function of perceived risk and benefit, cost and/or ability to pay, and demographic/social factors, including some unobserved effects. Accordingly, we included county-level dummy variables to capture unobserved heterogeneity and we clustered standard errors at the county level.

Our primary regression model specification includes an indicator for hard armoring on a neighbors parcel, geophysical characteristics (distance to the shoreline, elevation, slope, shoreline length, parcel area), shoreline change variables (indicators for medium- and high-energy environments, historical erosion rate), economic characteristics (building value), in addition to the county fixed effects. We tested for the influence neighborhood fixed effects (when information was available) to control for neighborhood-level hard armoring projects (that may be beyond the decisions of individual homeowners). Lastly, we estimated models without the neighboring parcel effect, in order to produce results that might be applied to other locations (under the expectation that neighboring armor indicator may not always be available). All of these results are compiled in the Appendix.

Final model selection was based primarily on variable influence, interpretability, ease of descriptor variable calculation, fidelity to processes supported by theory, and model fit diagnostics. Model prediction accuracy (“Accuracy” in the Appendix) is the in-sample prediction success, using a fitted value of 50% to predict armoring. To assess out-of-sample prediction and sensitivity to the cutoff value for armor prediction, we performed a 10-fold cross-validation and measured the area under the “Receiver Operating Characteristic” (ROC) curve to assess sensitivity and specificity. Sensitivity is the fraction of positive cases that are correctly classified by the logistic regression model, while specificity is the fraction of negative cases that are correctly classified. The ROC curve is the plot of sensitivity versus 1-specificity from a 10-fold cross validation, and area under the ROC (reported as “ROC auc” in Appendix) is commonly used as a measure of goodness of fit for out-of-sample prediction accuracy. We conducted likelihood ratio tests to assess nested model specifications and used Information Criteria for non-nested assessments. Lastly, we tested the final model for spatial autocorrelation assuming an inverse-distance weighting matrix.

## Results

**Figure 2:**
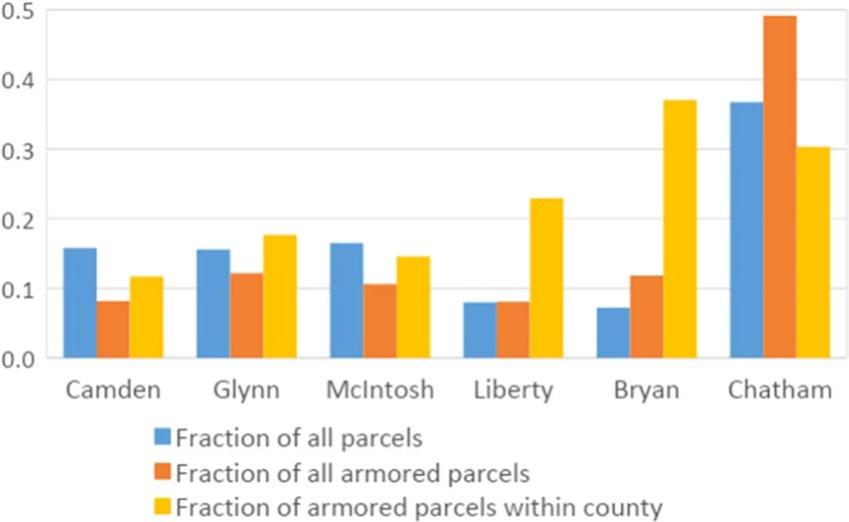
Fraction of all shoreline parcels (blue) and all armored shoreline parcels (orange) by county. Fraction of armored shoreline parcels within a county (yellow).

We identify 13,209 land parcels along Georgia’s estuarine shoreline for analysis; these parcels provide a census of armored and unarmored estuarine shoreline parcels. Chatham County comprises the largest number of these parcels (4856, 37%) and Bryan County comprises the fewest (955, 7%) (Fig. 2). In total, we estimate 2,997 parcels to be armored (23%). Chatham County also comprises the largest number of armored parcels (1473, 49%), whereas Liberty County comprises the fewest (242, 8%). When comparing armoring prevalence among counties, Bryan County has the highest percentage of armored parcels, with 37% of all estuarine shoreline parcels in Bryan County being armored (Fig. 2). In total, we estimate 4,004 parcels (30%) to be adjacent to a parcel with existing armoring.

### Logistic regression analysis

**Figure 3:**
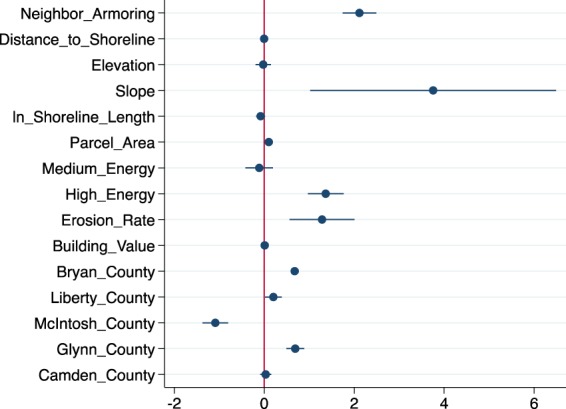
Forest plot of the change in the log-odds of the probability of hard armoring resulting from a unit increase in the predictor variables included in the logistic regression model. Positive values indicate a positive association with hard armoring likelihood and negative values indicate a negative association with hard armoring likelihood. Bars are 95% confidence intervals. Parameter intervals that overlap zero do not significantly influence the probability of hard armoring (at 5% significance level).

The final selected logistic regression model for describing patterns of hard armoring among estuarine shoreline parcels in Georgia includes the predictor variables shown in Fig. 3. A likelihood ratio test supported the inclusion of neighborhood fixed effects (Chi-square = 323.476, with 172 degrees of freedom), and this model exhibits a correct classification rate of 88%. Chatham County is used as the reference for county fixed effects. Structure value appears to be best represented by replacement cost per building area, which we term “building value” ($/m^2^). The explanatory power of shoreline length is improved by natural logarithm transformation. The urban classification descriptor variables (housing and population density) had inconsistent associations with armoring throughout model development, as well as small marginal effects and minimal influence on classification accuracy; thus, we did not include a measure of urban classification in the final model. The final model (Fig. 3) indicates that eight of ten landscape and socioeconomic attributes selected *a priori* are strong predictors of the log-odds of shoreline armoring (*p* < 0.1). (See Appendix A, column two for numerical regression results).

We find that parcel slope (elevation/distance from the shore) has the largest effect on the log-odds probability of hard armoring, with a change in the log-odds value of 3.75 and a marginal effect of 0.33. Thus, a one-unit increase in the slope (from an average of 0.025) increases the likelihood of armoring by 33%. More telling, however, the elasticity of slope is 0.03, indicating that a one-percent increase in slope increases the probability of armoring by only 0.03%. Distance from the shoreline, on its own, has a small negative effect on armoring (marginal effect of −0.0006), while elevation does not have a statistically significant effect (independent of slope).

Also very impactful in the logistic regression model, the “neighbor armoring” coefficient indicates a change in the log-odds average value of 2.32 and a marginal effect of 0.18. Thus, being located next to an armored parcel increases the likelihood of armoring by 18% (holding all other predictor variables constant). This effect may reflect environmental forcings that are common to all parcels in a particular area, spatial spillovers in erosion risk due to installation of hard armoring on neighboring properties, or herding behavior (in which landowners adopt practices they see their neighbors using). To attempt to control for this, we include indicators for medium-energy or high-energy shoreline environments (relative to low-energy) and the historical erosion rate. Model results suggest medium-energy environments have no discernable impact on armoring, but high-energy shoreline environments increase the likelihood of hard armoring by 12%. A one-unit increase in the historical erosion rate increases the probability of armoring by 11%.

Other predictor variables exhibited modest effects in the logistic regression model. A one-meter increase in shoreline length reduces the log-odds by 0.09 (the likelihood of hard armoring by 0.0002 – marginal effect not statistically significant). An additional square-meter of parcel area increases the log-odds of hard armoring by 0.105, with a marginal effect of 0.009. A one-dollar increase in structure replacement cost (per m^2^) increases the log-odds by 0.0093, with a marginal effect of 0.0008. Parcels located in Glynn, Liberty, and Bryan Counties are more likely to be armored, controlling for other predictors and neighborhood fixed effects, relative to Chatham County, while parcels in McIntosh County are less likely to be armored. Camden County was no different from Chatham County (all else being equal). The final model showed no evidence of spatial autocorrelation in errors (Moran’s I = 0.000246, *p* = 0.8125).

## Discussion

Nearly all of the hypothesized environmental and socioeconomic attributes are significantly predictive in a logistic regression model describing the distribution of hard armoring on estuarine shoreline parcels along the Georgia coastline. The model is generally intuitive and performs well, as indicated by a classification accuracy of 88% and a pseudo-R^2^ (McFadden’s) of 45.62%. The area under the ROC curve is 0.9028, indicating very good specificity and sensitivity in cross-validation. These findings suggest that a variety of interacting environmental and socioeconomic influences contribute to patterns of hard shoreline armoring (Appendix A).

The neighbor armoring variable has notably high influence on the likelihood of hard armoring; if a given parcel is adjacent to a parcel with hard armoring, the probability of the initial parcel having hard armoring increases by 18%. A large and statistically significant effect of neighboring shoreline armoring was also identified in survey results from 360 waterfront homeowners in Mobile Bay, Alabama. Survey analysis indicated that the condition of a neighboring shoreline (vertical wall vs. natural or revetment) was the most powerful explanatory variable in predicting a homeowner’s current shoreline condition (vertical wall, natural, or revetment); in that study, homeowners neighbored by a vertical wall had a >90% probability of also having a vertical wall^39^.

The mechanism behind the neighbor armoring influence is a subject that necessitates additional research, particularly due to the potential for a ‘snowball effect’ that could lead to widespread armoring^39^. One possible explanation for this phenomenon arises from the strong positive correlation between the presence of armoring and long-term historical erosion rates in our model. In particular, we find that parcels in high-energy environments are 12% more likely to be armored, and increasing the historical erosion rate by one meter per year increases the likelihood of armoring by 11%; this suggests that armoring emplacement is affected by flooding and erosion risk. It is reasonable to assume that armoring may cluster in areas, spanning multiple parcels along the shoreline that are naturally prone to highly erosive forces.

By modifying wave action and sediment flow, however, armoring can cause spatial externalities that lead to greater erosive forces on unarmored neighboring properties^49^. This effect has been identified in recent field reconnaissance along the Georgia coastline^50^, and this provides a second possible (not necessary mutually exclusive) explanation driving the neighbor armoring effect. A third potential driver is occurrence of herding behavior, in which parcel owners assume those who have installed armoring have well founded information supporting their decision and that their decision is the correct one^51^. Our analysis does not permit isolation of the mechanisms driving the neighbor armoring effect, but a more spatially inclusive fine-scale analysis of erosion factors and trends coupled with documentation of hard armoring structures and parcel owners motivations and perceptions would be valuable in attempting to further disentangle the influences underlying exogenous environmental factors, individual perceptions, and induced spatial effects on subsequent hard armoring decisions. Including fetch as a physical descriptor could also potentially improve the prediction accuracy of future models.

Considering topology of the coastline, we anticipated a negative correlation between elevation and hard armoring likelihood, but results do not support this. In isolation, elevation does not have a statistically significant effect on the likelihood of hard armoring, but slope (the ratio of elevation to distance to shoreline) has a strong positive effect on the probability of hard armoring. That is, a steep shoreline is more likely to have hard armoring (though given the low relief of the Georgia coast, the effect is not very large). Distance of the parcel centroid from the shoreline reduced the probability of armoring, but the effect was also small. While we were not able to observe building footprints, less depth of the parcel generally indicates that any improvements on the land will be closer to the shoreline, hence facing greater erosion and flood risk. We did include building value in the model, however, which we view as an indicator the benefits of erosion protection. Estuarine parcels with valuable capital assets are more likely to have shoreline armoring; this is consistent with the idea that hard armoring is a seen as a risk management investment as the return-on-investment is more favorable if the land parcel contains a valuable capital asset. Greater building value is also likely correlated with income and wealth, which increases ability to pay for hard armoring.

Parcel area has a significant positive association with armoring likelihood, indicating that larger properties are more likely to have hard armoring. We interpret this as an additional measure of benefit; larger parcels have greater amounts of land vulnerable to damage from erosion and/or flooding, thus increasing the benefits of protection. Similar to building value, larger parcels may be more commonly held by wealthier landowners with the ability to afford hard armoring. Shoreline length has a significant negative association with hard armoring. We interpret this as a measure of cost; longer shorelines may discourage hard armoring implementation, as costs increase with length.

Continuation of global mean SLR coupled with coastal population growth will directly affect the attributes included in our analysis, which should lead to evolving armoring probabilities over time. It is reasonable to propose that without large-scale regulatory intervention, the influential factors involved in the individual decision to erect hard armoring will remain similar into the near future, as recent studies show that hard armoring is continuing to be viewed as the most durable and effective form of coastal protection^16,39,52^. While our model is not explicitly dynamic, the positive association between the neighbor armoring attribute and armoring probability suggests the potential for widespread increases in hard armoring implementation in reaction to rising sea levels and increasing erosion rates.

Although future SLR response actions are ultimately uncertain, predicting evolutions in armoring distributions can provide insight potential impacts of armoring on ecological and socioeconomic systems, both current and future. In particular, we emphasize the need to consider evolving distributions of hard armoring in predictions of marsh sustainability through inland migration. With approximately 23% of parcels along Georgia’s estuarine shoreline already bounded by hard armoring structures, the inland migration potential of salt marshes is certain to be attenuated. Work to include dynamic future scenarios of hard armoring patterns in models predicting salt marsh response to SLR would result in a more accurate depiction of future habitat distributions as well as an understanding of the extent to which armoring decisions will affect salt marsh sustainability. Due to the complex nature of hard armoring decisions, the model we present here, that identifies both known and unknown variables influencing hard armoring decisions, provides a useful foundation for such an analysis.

The insights on hard armoring patterns provided by this study, including the indication of current or potential hot spots for armoring presence, will assist decision makers in designing planning efforts aligned with human and environmental tendencies. Management decisions will undoubtedly result in trade-offs between environmental stewardship and development interests; it is possible, however, that more efficient scenarios can be identified. For example, smart development and land-use planning could be informed by identification of areas better suited for facilitated marsh migration than development and vice-versa, as it is more feasible to aid marsh migration by setting aside land before it is developed than to remove development as sea level rises^53^.

While the 88% classification accuracy, supportive results from the 10-fold cross-validation, and highly significant predictor variables included in our model indicate a robust cross-sectional model for identifying general patterns of current estuarine hard armoring at the parcel scale, we acknowledge that our analysis cannot encompass all potential drivers of hard armoring emplacement. The study did not include interviews or surveys that could provide substantial insight into attitudes and perceptions that drive individual property owners’ decisions. As a result, we can only speculate as to what explains the relationship between neighbor hardening and parcel hardening. For example, hard armoring is commonly implemented for protection purposes, but may also be used to create a flat backyard or provide access to a boat ramp. Despite this caveat, our use of a census of armoring data at the parcel scale offers a novel and parsimonious approach to analyzing and improving understanding of patterns of hard shoreline armoring at scales relevant to management and policy-making. Our methodology can be readily adapted for management and engineering applications in other study areas, both with and without existing armoring data. We encourage further research to examine whether common predictors emerge across regions when consistent methods are used to analyze a census of shoreline parcels.

## Conclusions

Even if extreme efforts are taken to lessen human-induced climatic changes, global mean sea level is expected to continue to rise throughout the coming millennia^54,55^. Protecting dry land by constructing hard armoring has historically been the common societal response to SLR and this practice is likely to continue to proliferate into the near future. SLR response decisions made today will have profound and long-term impacts, many of which will disproportionately affect future generations^54^. While protecting dry land with hard armoring is associated with negative impacts such as coastal habitat and ecosystem services loss, there has been limited evaluation of the underlying drivers of this phenomenon.

This study advances the current understanding of hard shoreline armoring emplacement at the scale of individual landowners by documenting and describing patterns of hard armoring for a census of armored and unarmored estuarine shoreline parcels on the Georgia coastline. Our logistic regression model shows that a parsimonious set of socioeconomic and environmental attributes can be used to predict the presence of hard armoring with high accuracy. In particular, our model suggests that the presence of hard armoring on a neighboring parcel is one of the dominant factors in describing patterns of hard armoring. Also important are the historical erosion rate, energy level of the shoreline environment, and shoreline slope.

By analyzing a census of estuarine shoreline parcels in Georgia, the methodology used here is the first to provide a comprehensive, fine-scale characterization of estuarine shoreline hard armoring at a scope relevant to local and regional governments. With improved understanding of the drivers behind hard armoring implementation, as well as consideration of the associated current and future societal and environmental implications, this study enhances the opportunity to develop win-win scenarios through spatial planning and coincident management of development and coastal ecosystems.

## Supplementary information

Appendix A Shoreline armoring logistic regression models
