# Supplementary material for: Socioeconomic and environmental predictors of estuarine shoreline hard armoring
Source: Sci Rep. 2019 Nov 8;9:16288. doi: 10.1038/s41598-019-52504-y (PMC6841926; doi:10.1038/s41598-019-52504-y)
Supplement: Supplementary file 1 — Appendix A Shoreline armoring logistic regression models [file 41598_2019_52504_MOESM1_ESM.pdf]

Appendix A

Socioeconomic and environmental predictors of estuarine shoreline hard armoring

Nicole E. Peterson,[a,b] Craig E. Landry,[c] Clark R. Alexander,[d] Kevin Samples,[e] and Brian P. Bledsoe[f,*]

[a] Graduate Research Assistant, School of Environmental, Civil, Agricultural, and Mechanical Engineering, Institute for Resilient Infrastructure Systems (IRIS), University of Georgia, Athens, GA 30602
[b] EA Engineering, Science, and Technology, Inc., PBC, 320 Gold Avenue Southwest, Suite 1300, Albuquerque, NM 87102
[c] Professor, Department of Agricultural and Applied Economics, IRIS, University of Georgia, 0301 Conner Hall, 147 Cedar Street, Athens, GA 30602
[d] Director and Professor, Skidaway Institute of Oceanography, Department of Marine Science, IRIS, University of Georgia, 10 Ocean Science Circle, Savannah, GA 31411
[e] GIS Analyst, IRIS, School of Environmental, Civil, Agricultural, and Mechanical Engineering, University of Georgia, 0712L Boyd Graduate Research Building, 200 D.W. Brooks Drive, Athens, GA 30602
[f] Director and Professor, IRIS, School of Environmental, Civil, Agricultural, and Mechanical Engineering, University of Georgia, 0712M Boyd Graduate Research Building, 200 D.W. Brooks Drive, Athens, GA 30602; e-mail address: bbledsoe@uga.edu; Tel.: +1 706 542 7249; Fax: +1 706 542 2475.
[*] Corresponding Author

Table A.1. Shoreline armoring logistic regression models.

Variable	Model 1 NA / no NFE	Model 2 NA+NFE	Model 3 no NA / no NFE	Model 4 NFE / no NA
**arm_neigh**	2.3239***	2.1098***		
	(0.1452)	(0.1844)		
**distance**	-0.0023***	-0.0030***	-0.0038***	-0.0044***
	(0.0004)	(0.0004)	(0.0005)	(0.0006)
**elev**	0.0003	-0.0253	0.0428	0.0080
	(0.0618)	(0.0872)	(0.0864)	(0.1278)
**slope**	3.0852***	3.7527***	3.3221*	3.7672*
	(1.0270)	(1.4010)	(1.7471)	(2.1567)
**ln(shore_l)**	-0.0846**	-0.0914*	-0.1200**	-0.1381**
	(0.0430)	(0.0518)	(0.0609)	(0.0640)
**parcel_area**	0.0689*	0.1047**	0.1004**	0.1397***
	(0.0409)	(0.0421)	(0.0434)	(0.0463)
**med_energy**	-0.1170	-0.1057	-0.2245	-0.1119
	(0.1385)	(0.1548)	(0.1850)	(0.1809)
**hi_energy**	1.2610***	1.3607***	1.6908***	1.7695***
	(0.1872)	(0.1996)	(0.2461)	(0.2736)
**erosion_rate**	1.3069***	1.2841***	1.9639***	1.7726***
	(0.3963)	(0.3687)	(0.6569)	(0.6216)
**bldg_val**	0.0087***	0.0093***	0.0096***	0.0099***
	(0.0013)	(0.0012)	(0.0016)	(0.0015)
**bryan**	0.1124*	0.6761***	0.0181	0.6734***
	(0.0615)	(0.0252)	(0.0867)	(0.0452)
**liberty**	-0.3742***	-0.5709	-0.7354***	-0.7588
	(0.0634)	(0.5471)	(0.1176)	(0.6306)
**mcintosh**	-0.6610***	-1.0806***	-1.0225***	-1.0746***
	(0.0788)	(0.1405)	(0.1252)	(0.1590)
**glynn**	-0.0061	0.6286***	-0.0671	0.8140***
	(0.0827)	(0.1319)	(0.0945)	(0.1573)
**camden**	-0.5515***	-0.6814	-0.9414***	-0.8704
	(0.0330)	(0.5169)	(0.0677)	(0.5872)
**constant**	-2.4124***	-2.8219***	-1.0028***	-1.8569***
	(0.3144)	(0.3816)	(0.3699)	(0.4685)
**Observations**	13,209	13,209	13,209	13,209
**Neighb_FE**	NO	YES	NO	YES
**lnL**	-4008.493	-3846.755	-4806.206	-4436.230
**AIC**	8026.986	7703.510	9622.412	8882.459
**BIC**	8064.429	7740.953	9659.855	8919.902
**Pseudo R^2**	0.4333	0.4562	0.3205	0.3728
**Accuracy**	0.8736	0.8807	0.8458	0.8550
**ROC_auc**	0.9099	0.9028	0.8662	0.8724
	(0.0030)***	(0.0034)***	(0.0036)***	(0.0038)***

Standard errors in parentheses: *$p < 0.10$, **$p < 0.05$, ***$p < 0.01$.
NA is neighbor armoring variable; NFE is neighborhood fixed effects; "Accuracy" is the in-sample prediction accuracy; "ROC_auc" is the area under the "Receiver Operating Characteristic" curve, which is the plot of sensitivity versus 1-specificity from a 10-fold cross validation. (Sensitivity is the fraction of positive cases that are correctly classified by the logit model, while specificity is the fraction of negative cases that are correctly classified.)
